# A prospective cohort study on the association between cervical microenvironmental factors and the efficacy of treating high-risk human papillomavirus infection comorbid with cervical diseases

**DOI:** 10.3389/fcimb.2026.1734088

**Published:** 2026-03-10

**Authors:** Lingyun Ji, Jing Wu, Yang Zhou, Xiaowen Pu, Xiao Wang, Bowen Xu, Ruixian Jiao, Wenjuan Wu, Wenhong Zhang

**Affiliations:** 1 Department of Infectious Diseases, Shanghai Key Laboratory of Infectious Diseases and Biosafety Emergency Response, National Medical Center for Infectious Diseases, Huashan Hospital, State Key Laboratory of Genetic Engineering, School of Life Sciences, Fudan University, Shanghai, China; 2 Shanghai Sci-Tech Inno Center for Infection & Immunity, Shanghai, China; 3 Department of Laboratory Medicine, Shanghai East Hospital, Tongji University School of Medicine, Shanghai, China; 4 Department of Cervix, Shanghai Key Laboratory of Maternal Fetal Medicine, Shanghai Institute of Maternal-Fetal Medicine and Gynecologic Oncology, Shanghai First Maternity and Infant Hospital, School of Medicine, Tongji University, Shanghai, China; 5 Department of Traditional Chinese Medicine, Shanghai East Hospital, Tongji University School of Medicine, Shanghai, China; 6 The First Affiliated Hospital of Henan Polytechnic University, The Second People’s Hospital of Jiaozuo, Jiaozuo, China

**Keywords:** 16S rRNA gene sequencing, cytokines, high-risk human papillomavirus (HR-HPV), interferon-based local drug therapy, transcriptome

## Abstract

**Objective:**

Interferon-based local therapy is an intervention for high-risk human papillomavirus (HR-HPV)-associated low-grade squamous intraepithelial lesions (LSIL) or lower-grade cervical abnormalities. This study sought to delineate the differences in clinical outcomes following interferon-based local drug treatment and elucidate the microenvironmental factors driving these disparities.

**Methods:**

Cervical secretions, cell brush specimens, and cervical tissue samples were collected from patients with persistent HR-HPV infection and LSIL/lower-grade lesions at Shanghai First Maternity and Infant Hospital. Follow-up samples were obtained at 3 months post-treatment. Cervical secretions were subjected to 16S rRNA sequencing (to profile the microbiota) and cytokine quantification. Cell brush specimens were analyzed via transcriptome sequencing, while cervical tissue samples underwent immunohistochemical staining. Efficacy-related markers were assessed through both inter-group (independent comparisons) and intra-patient (self-paired) analyses.

**Results:**

At the transcriptome level, the HR-HPV clearance group exhibited lower enrichment in pathways related to differentiation, keratinization, and development but higher enrichment in immune activation pathways compared to the persistence group at baseline (with a reversed pattern observed at follow-up). Baseline expression of *TRAF3IP3, ZBP1*, and *IFI35* was higher in the clearance group, and *ZDHHC11* expression remained consistently elevated. Immunohistochemical findings further demonstrated that the percentage of TRAF3IP3- and ZBP1-positive cells at baseline was significantly higher in the clearance group than in the persistence group. At the microbial level, treatment failure was associated with reduced *Lactobacillus* abundance, increased *Gardnerella*, *Streptococcus anginosus*, *Schaalia turicensis*, and *Comamonadaceae* abundance, alongside higher alpha diversity. Among cervical secretory cytokines, IL-2, IL-8, IL-12p70 showed inter-group differences, while IL-4 and IL-5 were barely detectable.

**Conclusions:**

This study characterizes the cervical microenvironmental differences underlying divergent responses to interferon-based therapy, highlighting that coordinated changes in the microenvironment and immune status modulate treatment outcomes. The upregulated mRNA and protein levels of TRAF3IP3 and ZBP1 in the baseline period favor HR-HPV clearance, suggesting their potential as promising therapeutic targets.

## 1 Introduction

Human papillomavirus (HPV) infection is highly prevalent globally, primarily transmitted via sexual contact and intimate physical interaction. Notably, over 80% of women will experience at least one episode of HPV infection during their lifetime. In China, the incidence and mortality rates of cervical cancer remain among the highest worldwide. According to the 2023 ICO/IARC (International Papillomavirus Society/International Agency for Research on Cancer) Report on HPV-Associated Diseases in China, persistent infection with high-risk HPV subtypes is responsible for 98% of cervical cancer cases in Chinese women. Cervical cancer ranks as the third most common cancer (in terms of both incidence and mortality) among women in China, with approximately 109,741 new cases and 59,060 deaths attributed to the disease annually (Norenhag et al., 2020; China human papillomavirus and related cancers, fact sheet, 2023). Despite the availability of HPV vaccines, the burden of cervical cancer is projected to remain substantial over the next 30–50 years, driven by low immunization rates in low- and middle-income countries (LMICs) and inadequate global cervical cancer screening (Arbyn et al., 2020).

When the body is infected with high-risk HPV (HR-HPV), a robust immune system can typically clear the virus. A weakened immune system, however, may lead to persistent infection, which in turn progresses to cervical intraepithelial neoplasia (CIN). Based on lesion extent, CIN is classified into low-grade lesions (CIN1) and high-grade lesions (CIN2+). Early intervention for HPV-associated cervical lesions can prevent further progression (Zhang et al., 2025).

The human vaginal microbiome (VMB) plays a crucial role in sustaining vaginal health and homeostasis, while exhibiting lower diversity than the microbiomes of other bodily organs (Arbyn et al., 2020; Kyrgiou and Moscicki, 2022). Growing evidence indicates an association between the vaginal microbiome, human papillomavirus (HPV) infection, and cervical lesions (Ravel et al., 2011; France et al., 2020; France et al., 2022; Kwon and Lee, 2022; Kyrgiou and Moscicki, 2022; Shen et al., 2022; Zhu et al., 2022), Lactobacilli, on one hand, can reduce cervical epithelial cell permeability, mitigate inflammatory responses, and inhibit the proliferation of cervical cancer cells. Conversely, bacteria that increase vaginal microbiome (VMB) diversity may express genes related to cervical cell adhesion and cytotoxicity, thereby damaging cervical epithelial cells and contributing to human papillomavirus (HPV) infection and high-grade cervical lesions. Meanwhile, the HPV-encoded E7 protein can downregulate the secretion of defense peptides (which favor *Lactobacillus* growth) via the NF-κB and Wnt/β-catenin signaling pathways. This reduction in defense peptides elevates vaginal pH, further promoting the overgrowth of vaginal pathogens and ultimately inducing structural dysregulation of the VMB (Onderdonk et al., 2016). Regarding host-related factors, women with cervical lesions tend to inherently exist in a proinflammatory state, while host-derived antimicrobial peptides (AMPs) serve as key components of the mucosal immune barrier (Lebeau et al., 2022).

Previous studies have advanced HPV-related cervical cancer research across four key dimensions—HPV16 viral variant characteristics, viral invasion mechanisms, host molecular regulation, and non-coding RNA networks—with notable academic value: filling the genomic data gap of South American HPV16 variants by identifying cancer-associated SNPs (e.g., G145T, T350G) and high-risk Lineage D variants (de Paula Filho et al., 2024), clarifying the HPV16 L2 protein’s structure and its interaction with host S100A10 (Jiang et al., 2025), confirming ZNF695 as an independent prognostic biomarker and immunotherapeutic target (Ding et al., 2024), and revealing the regulatory role of the hsa_circ_0000021/miR-3940-3p/KPNA2 axis (Zeng et al., 2024). Collectively, these findings provide critical theoretical support for regional precision prevention, antiviral drug development, and precision diagnosis/treatment, laying a solid foundation for advancing basic research and therapeutic innovation in HPV-related cervical cancer.

Interferon-based topical therapy is a relatively conservative treatment option for patients with low-grade cervical lesions (LSIL) or lower-grade abnormalities, with an HPV clearance rate of approximately 30–90% (He et al., 2022). The therapeutic intervention used recombinant human interferon α-2b vaginal effervescent capsules (trade name: xinfuning, 800,000 IU/capsule) for local cervical administration. Patients self-administered one capsule intravaginally nightly (posterior fornix, near cervical os), with 12 consecutive days of monthly treatment and suspension during menstruation. The total course was 3 months, with monthly follow-up for safety evaluation and subsequent medication dispensing. Its mechanism of action involves activating the JAK/STAT signaling pathway to inhibit viral transcription, translation, and nucleic acid replication, thereby exerting antiviral effects (Bekisz et al., 2010).

Given that vaginal microbiota and host factors are known to influence HPV infection, we hypothesize that these two factors may contribute to the observed variability in interferon efficacy. Currently, the reasons underlying differential treatment responses—i.e., why some patients achieve HPV clearance following topical therapy while others remain persistently infected—remain unclear. Thus, this study aims to analyze differences in clinical outcomes after interferon-based topical treatment for HPV-associated cervical disease and provide evidence-based guidance for tailored pharmacotherapy in HPV-associated gynecological conditions.

## 2 Materials and methods

### 2.1 Research subjects

Cervical secretions, cell brushes and cervical tissue of patients with high-risk HPV persistent infection and pathological results of low-grade lesions or below were collected from the Department of Cervix of Shanghai First Maternity and Infant Hospital, Follow-up samples except cervical tissue were collected at the 3-month reexamination after interferon-based local therapy. In this study, persistent HR-HPV infection was defined as two positive results for the same high-risk HPV genotype with an interval of at least 6 months between the two tests (confirmed via preliminary examinations and interviews) and enrolled patients were administered a standardized 3-month treatment. Inclusion criteria:(a) Regular menstrual cycles (25–35 days); (b) Age 18–49 years; (c) Non-pregnant and non-menstrual at sampling. Exclusion criteria: (a) Vaginal irrigation or medication within 3 days; (b) Receiving immunosuppressive therapy. This study was approved by the Medical Ethics Committees of Shanghai First Maternity and Infant Hospital (Ethics No.: KS 23359 and Ethics No.: KS 23353).

### 2.2 Sample collection

Cervical secretions: Samples were collected during non-menstrual periods using a dry sterile cotton swab from the cervical orifice. Within 4 hours, secretions were eluted in 1 mL of sterile normal saline (in a sterile EP tube), vortexed, and centrifuged at 4000 rpm at 4 °C for 10 minutes. The supernatant was stored at -80 °C for cytokine detection, and the precipitate was reserved for 16S rRNA sequencing.

Cervical cell brushes: Specimens were immersed in PreservCyt solution (liquid cytology vial) and frozen at -80 °C for subsequent transcriptome analysis and RNA extraction (for RT-PCR validation).

Cervical tissue specimens: Patients were placed in the lithotomy position with bladder emptying. The vulva, vagina, and cervix were disinfected with iodophor, followed by gentle insertion of a vaginal speculum to retract the vaginal walls and fully expose the cervix. The cervical surface was wiped with sterile gauze to remove excess secretions. For suspected lesions, a small tissue fragment (approximately 2–3 mm in diameter) was excised from the target area using biopsy forceps. Tissues were immediately placed in RNAlater, snap-frozen in liquid nitrogen, and stored at -80 °C until use.

### 2.3 Detection of 12 cytokines (immunofluorescence assay)

Flow cytometry was performed using a Mindray BriCyteE6. A cytokine panel kit (Shanghai Yingyu Biotechnology) was used, based on immunofluorescence staining: 12 capture microspheres (with specific antibodies) bound to sample cytokines, then to PE-labeled detection reagents, forming a double-antibody sandwich complex. Cytokine levels were determined by fluorescence intensity (to assess immune function). Unqualified samples (e.g., hemolyzed) were excluded. Wilcoxon test for intra-individual paired comparisons; Mann-Whitney for independent comparisons (significance: P<0.05).

### 2.4 16S rRNA gene sequencing

#### 2.4.1 Wet lab procedures

Total DNA was extracted using the E.Z.N.A.^®^ Soil DNA Kit (Omega Bio-tek). Concentration/purity was measured by NanoDrop2000; quality by 1% agarose gel electrophoresis. The V3-V4 region was amplified via PCR using primers 338F (5 ‘-ACTCCTACGGGAGGCAGCAG-3’) and 806R (5 ‘-GGACTACHVGGGTWTCTAAT-3’). PCR products were recovered (2% agarose gel), purified (AxyPrep DNA Gel Extraction Kit), eluted (Tris-HCl), and quantified (Qubit4.0). Illumina libraries were constructed, followed by PE300 sequencing (Illumina MiSeq platform).

#### 2.4.2 Dry lab procedures

Statistical analysis and visualization were performed in R (packages: vegan v2.6-4, phyloseq v1.38.0, tidyverse v1.3.2, ggpubr v0.5.0, ComplexHeatmap v2.10.0, corrplot v0.92). Alpha diversity was estimated using the PD, Observed, ACE, Chao1 indices. Inter-group microbial differences were analyzed via nonparametric rank sum test; species correlations via Spearman rank correlation. P-values were corrected using the BH method (significance: P<0.05). Linear discriminant analysis (LDA) effect size (LEfSe) method was performed with a p-value < 0.05 for the Kruskal–Wallis test and a size-effect threshold of 2.0 on the logarithmic LDA score. The functional prediction analysis of the cervical microbiome, implemented via PICRUSt2.

### 2.5 Transcriptome sequencing

#### 2.5.1 Wet lab procedures

Cervical cell brush specimens were sent to Honsunbio Technology Co., Ltd (Shanghai, China) for total RNA extraction (TransZol up, TransGen Biotech). mRNA was enriched via Oligo(dT) magnetic beads. Libraries were constructed (VAHTS Universal V8 RNA-seq Library Prep Kit for Illumina) and quantified (Qubit4.0). PE150 sequencing was performed on the Illumina Novaseq platform.

#### 2.5.2 Dry lab procedures

Expression analysis was done using StringTie; differential expression via EdgeR. P-values for the screened differentially expressed biomarker genes were adjusted by the BH method (P<0.05 for significance). GO/KEGG pathway enrichment was analyzed using clusterProfiler (Fisher’s exact test).

### 2.6 RT-qPCR

We validated selected immune-related genes of interest. Specifically, we amplified *TRAF3IP3*, *ZBP1*, *IFI35*, and *ZDHHC11* using gene-specific primer sequences. Total RNA was extracted and purified with the RNA Isolation Kit (Vazyme) following the manufacturer’s protocols. Reverse transcription and quantitative PCR (qPCR) were performed using Takara reagents. The primer sequences used for qPCR are provided in **Table 1**. All experiments were run in triplicate, and threshold cycle (Ct) values were determined using the Roche LightCycler^®^ 480 Instrument Sequence Detection System. Relative mRNA expression levels in each sample were calculated using the ΔΔCt method. Statistical analyses were conducted using the Wilcoxon test for intra-individual paired comparisons and the Mann-Whitney test for independent group comparisons, with statistical significance set at P < 0.05.

**Table 1:** Primer information for RT-PCR validation assays.

Genes	Forward primer (5’ to 3’)	Reverse primer (5’ to 3’)
*GAPDH* (Lebeau et al., 2022)	ACCAGGTGGTCTCCTCTGAC	TGCTGTAGCCAAATTCGTTG
*TRAF3IP3* (Deng et al., 2020)	TTCTCCCAGAGAGCAGGTGA	TGGTGTTTGGGTGGCTTCTT
*ZBP1*	CCATTGCAAACTCCGAAGCC	CCAGGGATCAACTAGGGTCC
*IFI35*	CAGGTGATGATGTCCAGCCA	CCTAGCAAACCCCAGCATGA
*ZDHHC11* (Liu et al., 2018)	TCATCCCCTTCCCGTGCCGT	CGCCCTGGGCTCATCTGCAC

### 2.7 Immunohistochemical analysis

After sectioning the tissues on ice, immunohistochemical staining was performed for four markers: TRAF3IP3, ZBP1, IFI35, and ZDHHC11, using primary antibodies purchased from Abmart Shanghai Co., Ltd. All immunolabeled sections were independently evaluated by experienced histopathologists. For quantification, each tissue section was examined at 50× magnification, and the proportion of immunopositive cells was calculated using ImageJ software.

### 2.8 Technical route

See **Figure 1**.

**Figure 1:**
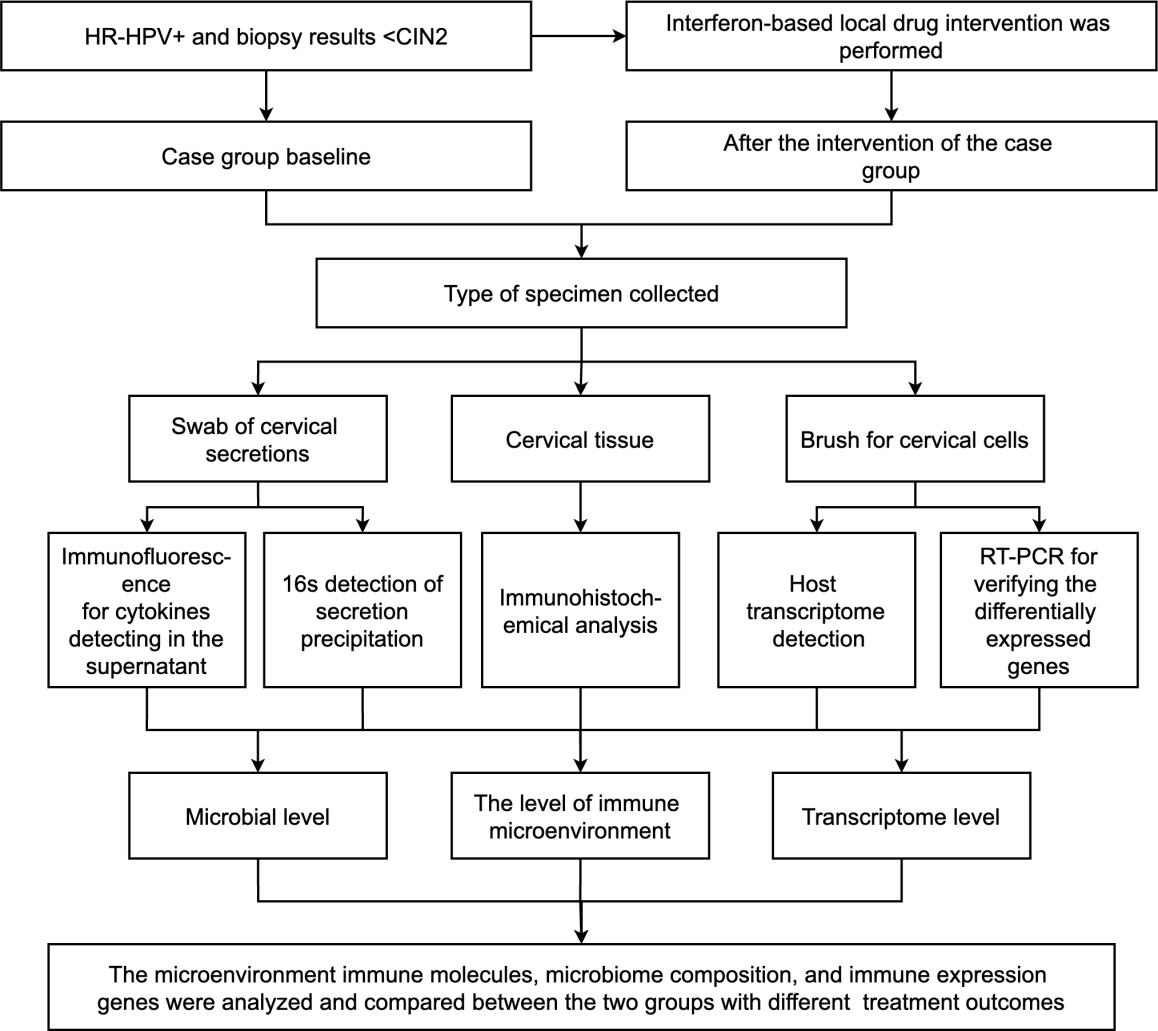
Technical route of the study.

## 3 Results

### 3.1 Clinical data characteristics

Baseline clinical data of patients with different treatment outcomes are shown in **Table 2**. No statistically significant differences were observed between the Clearance and Persistence groups before therapy.

**Table 2:** Clinical data of the baseline status of the subjects.

Variables	Clearance group	Persistence group	P value
Cytokine /16s/ transcriptome	10	19	
Age	36.9 ± 5.280	33.84 ± 5.480	0.1598*
HPV infection status			
Multiple HPV infections	1/10 (HPV52,HPV44)	1/19 (HPV16,HPV42)	>0.9999
HPV52 Single infection	4/10	7/19	>0.9999
HPV16 Single infection	0/10	0/19	>0.9999
HPV18 Single infection	2/10	2/19	0.592
Other HR-HPV Single Infection	3/10	9/19	0.4495
Duration of HR-HPV infection	10/10 (6–12 months)	19/19 (6–12 months)	
Colposcopy results			
Negative	1/10	1/19	>0.9999
Inflammation	7/10	16/19	0.6328
LSIL	2/10	2/19	0.592
TCT results			
Negative	2/10	10/19	0.1261
Ascus	3/10	3/19	0.6328
LSIL	2/10	3/19	>0.9999
Not checked	3/10	3/19	0.6328

### 3.2 Independent comparisons: cervical microenvironmental factors influencing treatment outcomes

#### 3.2.1 Baseline comparisons (clearance-B group vs. persistence-B group)

**Figure 2:**
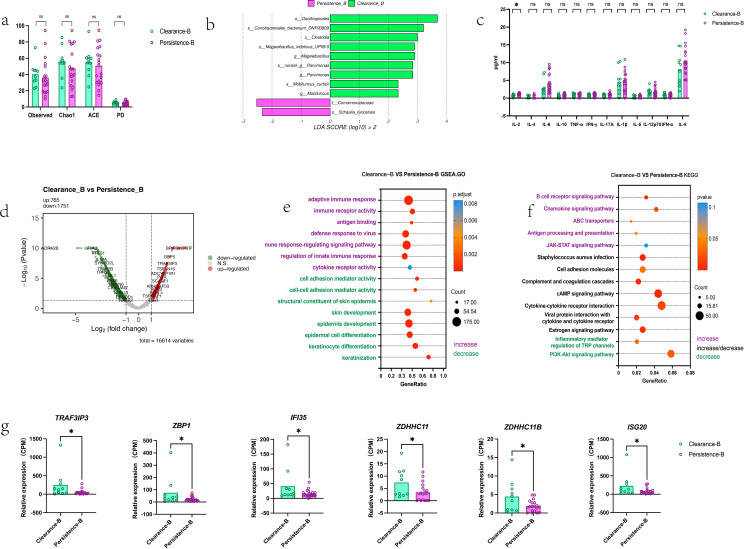
Baseline cervical microenvironmental factors influencing topical interferon therapy outcomes in high-risk HPV-infected patients **(a)** Baseline cervical microbial alpha diversity: Comparison between the two groups(unpaired t test) **(b)** Baseline bacterial enrichment (LEfse analysis): Persistence-B group vs. Clearance-B group **(c)** Baseline cervical secretion cytokines: Clearance-B group vs. Persistence-B group (*Note: Data represent the third root of original concentrations to standardize 12 cytokines, consistent with the statistical results of between-group comparisons using raw data, Mann-whitney).***(d)** Baseline host differentially expressed genes: Clearance-B group vs. Persistence-B group **(e)** Baseline upregulated pathways (GSEA enrichment): Clearance-B group vs. Persistence-B group **(f)** Baseline upregulated pathways (KEGG enrichment): Clearance-B group vs. Persistence-B group **(g)** Baseline interferon-related upstream and downstream gene expression: Clearance-B group vs. Persistence-B group, *Mann-whitney*. Statistical significance was defined as P < 0.05 (ns, not significant; * P < 0.05). All authors approve this clarification of the statistical symbols.

**Figure 2** illustrates the baseline cervical microenvironmental factors influencing the efficacy of local interferon therapy in patients with HR-HPV infection. As shown in the Figure, no statistically significant differences in alpha diversity were observed between the clearance and persistence group at baseline (**Figure 2a**). Baseline levels of *s-Schaalia turicensis* and *f-Comamonadaceae* were higher in the persistence group (**Figure 2b**). Furthermore, results of the functional prediction analysis of the cervical microbiome revealed that pathways such as carbon fixation in photosynthetic organisms were upregulated in the clearance group, whereas pathways including fructose and mannose metabolism were upregulated in the persistence group (**Supplementary Figure 1a**). Cytokine detection in cervical secretions showed no significant differences between the clearance and persistence groups, except for IL-2 (**Figure 2c**). Besides, A subset of key pro-inflammatory cytokines (IL-6, IL-10, IFN-γ and IL-8) in cervical secretions showed a trend of higher mean levels in the persistence group than in the clearance group, though no statistically significant differences were detected between the two groups for all measured cytokines (**Supplementary Figure 2**). At the transcriptomic level, the clearance-B group exhibited a greater number of differentially expressed genes (DEGs) compared with the persistence-B group; specifically, 765 genes were upregulated and 1751 genes were downregulated in the clearance-B group relative to the persistence-B group (**Figure 2d**). Gene Set Enrichment Analysis (GSEA) results showed enrichment of pathways associated with virus defense and immune response in the clearance group, while the persistence group had enrichment of cell development, differentiation and keratinization (**Figure 2e**). KEGG enrichment analysis of these DEGs revealed that immune-related pathways were predominantly enriched in the clearance group. The Jak-stat pathway, though not statistically significant, was enriched in the clearance group (**Figure 2f**). In addition, we found that the baseline expression levels of genes associated with differentiation, keratinization, and development, were lower in the clearance group than in the persistence group, examples included *KLK14, EMP1, SPRR2G, FABP5, SPRR1A, LCE3A, LCE3E, KRT14, KRTDAP, LOR, SPRR2E* etc. (**Supplementary Table 1**). Furthermore, baseline overall expression of immune-associated genes was higher in the clearance group compared with the persistence group, examples included *IFI35, ACOD1, DDX58, OAS3, GBP1, ZBP1, RSAD2, DDX60, C3AR1, TRAF3IP3, ISG20, ZDHHC11, ZDHHC11B* etc. (**Supplementary Table 1**). Furthermore, we investigated the role of host immune regulatory genes in treatment outcomes and found that baseline expression of interferon production related upstream genes including *TRAF3IP3, ZBP1, IFI35, ZDHHC11, ZDHHC11B* and downstream gene *ISG20* was higher in the clearance group than in the persistence group (**Figure 2g**). This implies baseline activation of interferon-related immunity in the clearance group.

#### 3.2.2 Follow-up comparisons (clearance-A group vs. persistence-A group)

**Figure 3:**
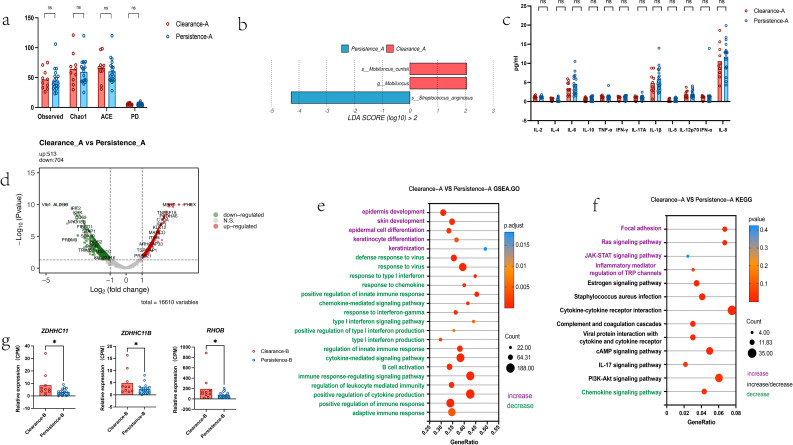
Differences in cervical microenvironment between high-risk HPV-infected patients with different outcomes at 3-month follow-up after topical interferon therapy (n=29) **(a)** Cervical microbial alpha diversity during follow-up: Clearance-A group vs. Persistence-A group (unpaired t test) **(b)** Bacterial enrichment (LEfse analysis) during follow-up: Persistence-A vs. Clearance-A group group **(c)** Cervical secretion cytokines during follow-up: Clearance-A group vs. Persistence-A group (*Note: Data represent the third root of original concentrations to standardize 12 cytokines, consistent with the statistical results of between-group comparisons using raw data., Mann-whitney)***(d)** Host differentially expressed genes during follow-up: Clearance-A group vs. Persistence-A group **(e)** Upregulated pathways (GSEA enrichment) during follow-up: Clearance-A group vs. Persistence-A group **(f)** Upregulated pathways (KEGG enrichment) during follow-up: Clearance-A group vs. Persistence-A group **(g)** Immune-related gene expression during follow-up: Comparison between the two groups, *Mann-whitney*.

Twenty-nine patients with high-risk HPV infection were followed up 3 months post local interferon treatment. Differences in the cervical microenvironment between the clearance and persistence groups are presented in **Figure 3**. No significant differences in alpha diversity were found between the clearance and persistence groups during follow-up (**Figure 3a**), however, *Streptococcus anginosus* was enriched in the persistence group (**Figure 3b**). Furthermore, no significant differences were found in 12 cytokines levels in cervical secretions between the two groups (**Figure 3c**). Relative to the persistence group, the persistence group had 513 upregulated and 704 downregulated genes (**Figure 3d**). Follow-up GSEA results showed enrichment of pathways associated with cell development, differentiation and keratinization in the clearance group and virus defense and immune response in the persistence group—contrary to the baseline GSEA findings (**Figure 3e**). Follow-up KEGG pathway enrichment analysis revealed that chemokine signaling pathways were enriched in the persistence group. Notably, JAK-STAT signaling pathway was persistently enriched in the clearance group but not statistically significant (**Figure 3f**). Furthermore, follow-up analysis showed higher expression of differentiation, keratinization, and development associated genes including *KLK14, EMP1, SPRR2G, KLK5, CALML5, LCE3E, KRTDAP* etc. (**Supplementary Table 2**). in the clearance group than in the persistence group. These genes (with the exception of *KLK5* and *CALML5*) were previously referenced in the baseline analysis, and their follow-up expression pattern was opposite to that at baseline. Furthermore, follow-up analysis showed lower overall expression of immune-related genes including *ACOD1, ZBP1, RSAD2, C3AR1* etc. (**Supplementary Table 2**). in the clearance group than in the persistence group, which previously analyzed in the baseline, exhibited an expression pattern contrary to the baseline comparison results. We found that *ZDHHC11* and *ZDHHC11B* were highly expressed in the clearance group during follow-up, which was consistent with the baseline results, furthermore, RHOB exhibited higher expression in the clearance group than persistence group during follow-up (**Figure 3g**).

### 3.3 Self-paired comparisons: cervical microenvironmental factors influencing treatment outcomes

#### 3.3.1 Overall paired comparisons (29 patients)

**Figure 4:**
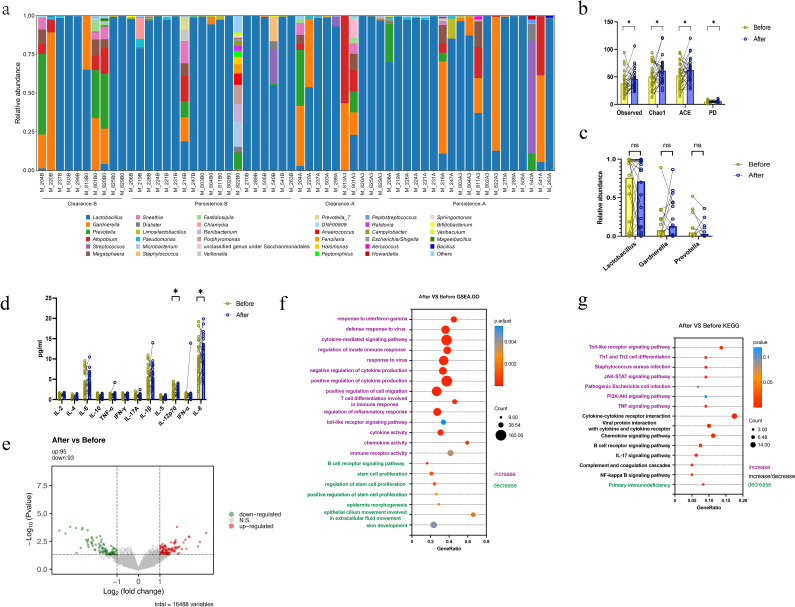
Paired comparisons of cervical microbiome, host transcriptome, and microenvironmental factors (cytokines) before vs. after treatment between the two groups **(a)** Overall cervical microbial composition of 29 paired specimens (58 total), stratified into 4 subgroups: Clearance-B group, Persistence-B group, Clearance-A group, Persistence-A group **(b)** Overall treatment-related changes in microbial diversity (paired t test) **(c)** Overall treatment-related changes in bacterial distribution (Wilcoxon matched-pairs signed rank test) **(d)** Overall treatment-related changes in cytokines (Wilcoxon matched-pairs signed rank test) *Note: To standardize data for 12 cytokines, results represent the third root of original measured concentrations, consistent with the statistical results of between-group comparisons using raw data*. **(e)** Paired comparisons of differential gene expression changes between the two groups after overall treatment **(f)** Paired comparisons of pathway changes (GSEA enrichment) between the two groups after overall treatment **(g)** Paired comparisons of pathway changes (KEGG enrichment) between the two groups after overall treatment.

We present the composition of the cervical microbiota in the 29 patients before and after treatment, which provides a visual overview of the changes and illustrated in (**Figure 4a**). In 29 patients undergoing topical interferon-based therapy, overall alpha diversity increased post-treatment (**Figure 4b**), while no significant differences were found in *Lactobacillus*, *Gardnerella* and *Prevotella* (**Figure 4c**). Results from the functional prediction analysis of the cervical microbiome revealed that retinol metabolism were upregulated in the pre-treatment group, whereas pathways including ascorbate and aldarate metabolism were upregulated in the post-treatment group (**Figure 4d**). (**Supplementary Figure 1b**). Post-treatment levels of IL-12p70 and IL-8 in cervical secretions were higher than pre-treatment levels. Several cytokines such as IL-2, IL-4, IL-10, TNF-α, IFN-γ, IL-17A, IL-5 showed low levels in the cervical microenvironment, notably, IL-4 and IL-5 were extremely low, while IL-6, IL-8, IL-1β and other cytokines were highly expressed (**Supplementary Table 3**). At the transcriptome level, pairwise comparisons of the 29 patients (pre- vs. post-treatment) identified few DEGs, with 95 up-regulated and 93 down-regulated genes after treatment (**Figure 4e**). GSEA revealed upregulated pathways following treatment, such as the response to virus. (**Figure 4f**). KEGG enrichment analysis of differential genes indicated enhanced overall cervical immune response post-treatment, which was manifested by the enrichment of Jak-stat pathway (**Figure 4g**).

#### 3.3.2 Group-specific paired comparisons (clearance vs. persistence groups)

**Figure 5:**
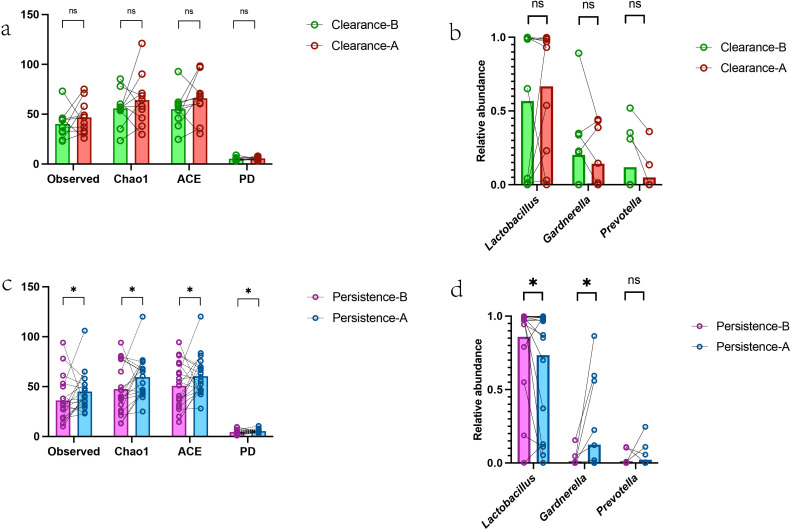
Changes in cervical microenvironment of subjects with different interferon treatment outcomes **(a)** Paired comparison of cervical microbial diversity changes in the Clearance group (Clearance-B vs. Clearance-A, Paired t test) **(b)** Paired comparison of cervical bacterial species distribution changes in the Clearance group (Clearance-B vs. Clearance-A, Wilcoxon matched-pairs signed rank test) **(c)** Paired comparison of cervical microbial diversity changes in the Persistence group (Persistence-B vs. Persistence-A, Paired t test) **(d)** Paired comparison of cervical bacterial species distribution changes in the Persistence group (Persistence-B vs. Persistence-A, Wilcoxon matched-pairs signed rank test). Statistical significance was defined as P < 0.05 (ns, not significant; * P < 0.05). All authors approve this clarification of the statistical symbols.

After overall post-treatment analysis, we compared intra-individual cervical microbiota changes between the clearance and persistence groups (post local interferon therapy) to explore outcome-related differences. No significant changes in alpha diversity were observed in the clearance group (**Figure 5a**), while alpha diversity increased significantly in the persistence group (**Figure 5c**). Post topical interferon treatment, specific microbial species showed no significant inter-group difference, but the clearance group had increased in *Lactobacillus* and decreased *Gardnerella* and *Prevotella* (**Figure 5b**), while the persistence group had the opposite (decreased *Lactobacillus*, increased *Gardnerella* and *Prevotella*) (**Figure 5d**). Results from the functional prediction analysis of the cervical microbiome revealed the clearance group additionally displayed significant changes in pathway of valine, leucine and isoleucine degradation (**Supplementary Figure 1c**) while persistence group in streptomycin biosynthesis etc. (**Supplementary Figure 1d**). At the microbial level, differential outcomes correlate with the distinct trends of alpha diversity and the dominant bacteria.

**Figure 6:**
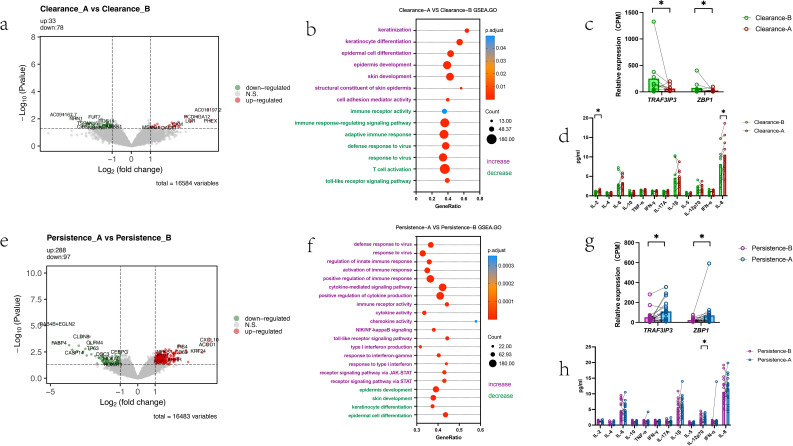
Transcriptome changes in cervical microenvironment after interferon treatment, and genes/cytokine differences by treatment outcome **(a)** Clearance group: Paired comparison of differential gene expression (Clearance-A vs. Clearance-B) **(b)** Clearance group: Pathway changes (Clearance-A vs. Clearance-B) **(c)** Clearance group: Specific gene changes (Clearance-B vs. Clearance-A, Wilcoxon matched-pairs signed rank test) **(d)** Clearance group: Cytokine changes (Clearance-B vs. Clearance-A) *Note: To standardize data for 12 cytokines, results represent the third root of original measured concentrations, consistent with the statistical results of between-group comparisons using raw data.* Wilcoxon matched-pairs signed rank test) **(e)** Persistence group: Paired comparison of differential gene expression (Persistence-A vs. Persistence-B, Wilcoxon matched-pairs signed rank test) **(f)** Persistence group: Pathway changes (Persistence-A vs. Persistence-B) **(g)** Persistence group: Specific gene changes (Persistence-B vs. Persistence-A, Wilcoxon matched-pairs signed rank test) **(h)** Persistence group: Cytokine changes (Persistence-B vs. Persistence-A, Wilcoxon matched-pairs signed rank test). Statistical significance was defined as P < 0.05 (ns, not significant; * P < 0.05). All authors approve this clarification of the statistical symbols.

We further analyzed host-level differences in treatment outcome-related changes between the two groups via intra-individual (pre- vs. post-treatment) comparisons. The clearance group had 33 up-regulated and 78 down-regulated DEGs post-treatment (**Figure 6a**), while the persistence group had 288 up-regulated and 97 down-regulated DEGs (**Figure 6e**). GSEA functional enrichment showed differential post-treatment patterns: the clearance group had upregulated the cell development, differentiation and keratinization function and downregulated antiviral immunity (**Figure 6b**), while the persistence group had downregulated the cell development, differentiation and keratinization function and upregulated antiviral immunity (**Figure 6f**). We further found post-treatment, *TRAF3IP3* and *ZBP1* were downregulated in the clearance group but upregulated in the persistence group (**Figures 6c, g**). Results of the 12 cytokines (secretory samples) showed increased IL-2 and IL-8 levels in the clearance group post-treatment (**Figure 6d**), while the persistence group had a significant change in IL-12p70 (**Figure 6h**).

### 3.4 Internal and external verification results

**Figure 7:**
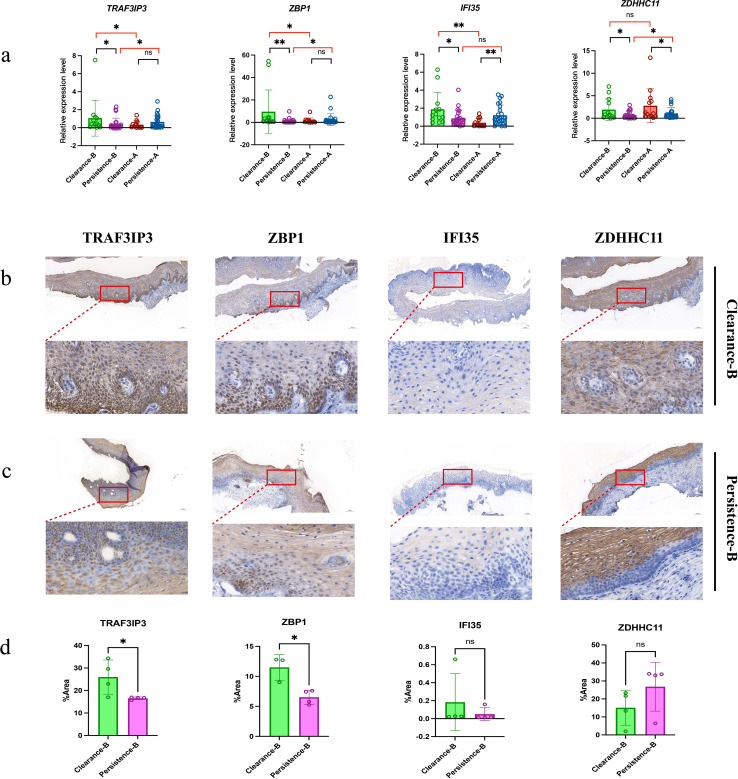
Differential expression and immunohistochemical analysis of TRAF3IP3, ZBP1, IFI35, and ZDHHC11 in cervical samples. **(a)** Relative mRNA expression levels of TRAF3IP3, ZBP1, IFI35, and ZDHHC11 across different groups. Statistical analyses were performed using the Wilcoxon test for intra-individual paired comparisons and the Mann-Whitney test for independent group comparisons; statistical significance was defined as P < 0.05 (ns, not significant; * P < 0.05; ** P < 0.01). **(b)** Representative immunohistochemical staining of TRAF3IP3, ZBP1, IFI35, and ZDHHC11 in cervical tissues from the Clearance-B group. Images include low-magnification views (upper panel) and corresponding high-magnification insets (lower panel) to clearly illustrate the distribution and staining intensity of immunopositive cells. **(c)** Representative immunohistochemical staining of TRAF3IP3, ZBP1, IFI35, and ZDHHC11 in cervical tissues from the Persistence-B group. Images are presented with low-magnification views (upper panel) and high-magnification insets (lower panel) following the same format as **(b)**. **(d)** Quantitative analysis of the proportion of immunopositive cells for TRAF3IP3, ZBP1, IFI35, and ZDHHC11 in cervical tissues from the Clearance-B and Persistence-B groups. Statistical comparisons were conducted to assess inter-group differences, with significance set at P < 0.05.

Next, we performed validation of selected immune-related genes of interest: first, we extracted RNA from the residual cervical brush specimens of the 29 participants, in addition, we collected 9 paired pre- and post-treatment samples from patients, comprising 3 patients in the clearance group and 6 patients in the persistence group. and subsequently employed real time-polymerase chain reaction (RT-PCR) to conduct validation of our transcriptome sequencing results. We utilized the primer sequences for the amplification of *TRAF3IP3, ZBP1, IFI35, ZDHHC11*. It can be seen that the results of RT-PCR validation for the four genes were consistent with those from the transcriptomic analysis (**Figure 7a**). In addition, we selected 8 tissue samples of the clearance group and the persistence group in the baseline period for immunohistochemical analysis of 4 indicators, and we selected 1 case of the clearance group in the baseline period as a representative figure shown in **Figure 7b**. We selected one case of persistence group in the baseline period as a representative figure shown in **Figure 7c**. Immunohistochemical results of eight specimens in clearance group and persistence group were quantitatively analyzed, and the results are shown in **Figure 7d**. It can be seen that the expression of TRAF3IP3 and ZBP1 in the clearance group was higher than that in the persistence group at the baseline protein level, which was consistent with the mRNA level of these two markers, while IFI35 seemed not to be expressed at the protein level, showing a negative result. ZDHHC11 was strongly expressed in the two groups, but there was no statistical difference.

## 4 Discussion

Cervicovaginal microbiome (CVM) features correlate with high-risk HPV (HR-HPV) progression. Prior studies indicate that Gardnerella-associated CIN2+ progression may be mediated by elevated cervicovaginal bacterial diversity. Microbiome signatures linked to HR-HPV progression could serve as therapeutic targets to prevent CIN2+ (Usyk et al., 2020). Our study supports this idea: local interferon therapy clearance group showed no significant change in microbial diversity between visits, while persistence group had increased diversity—along with reduced dominant *Lactobacillus* and elevated *Gardnerella*—changes not observed in clearance group. This study reconfirms that topical drug treatment failure correlates with increased individual microbial richness, reduced dominant *Lactobacillus*, and elevated pathogenic genera such as *Gardnerella.* At baseline, *Schaalia turicensis* and *f-Comamonadaceae* were more frequently detected in persistence group than clearance group. Both are previously reported as potential pathogens of genital infections (Nokchan et al., 2021). During follow-up, *Streptococcus anginosus* was more frequently detected in the persistence group than in the clearance group. Studies indicate that human papillomavirus (HPV) and oral bacteria *Streptococcus anginosus* are infectious risk factors for HPV-associated oropharyngeal squamous cell carcinoma (HPV-positive OPSCC) (Robayo et al., 2019). Our results further validate that increased non-*Lactobacillus* flora is associated with poor therapeutic outcomes.

Transcriptome analysis showed at baseline viral defense and immune response functions enriched in HPV-clearance patients while cell development, differentiation and keratinization functions low. At follow-up, patterns reversed with immune response lower in HPV-clearance patients than HPV-persistence patients, cell development, differentiation and keratinization functions higher in HPV-clearance patients. This indicates clearance group had baseline immune activation, which likely contributed to their favorable treatment outcomes. Additionally, JAK-STAT signaling pathway was consistently enriched in clearance group at baseline and follow-up, though the difference was not statistically significant. Prior studies on the transcriptomic landscape of HR-HPV-infected stratified epithelia showed epithelial “hidden cells” form compartments and may mark HPV-infected tissues and *ELF3* is the transcriptional driver required for HPV-induced hidden compartments (Bedard et al., 2023). Our transcriptome data revealed a correlation between keratinization function and therapeutic efficacy, highlighting its potential as a future antiviral treatment target. Pre-treatment immune activation is key for successful viral clearance. We analyzed specific IFN production related genes including *TRAF3IP3, ZBP1, IFI35, ZDHHC11, ZDHHC11B*, and the effector gene *ISG20*. At baseline, the clearance group showed significantly stronger interferon (IFN) production and responsiveness than the persistence group—this is one key reason for their ability to clear the HR-HPV. Another key finding is that *ZDHHC11* and *ZDHHC11B* were consistently highly expressed in the clearance group across both periods. ZDHHC11 is a protein acyltransferase that can regulate downstream genes or signaling pathways through palmitoylation modification and other ways (Pei and Piao, 2024). *ZDHHC11B* and *ZDHHC11* are highly homologous palmitoyltransferase family members acting synergistically in lipid metabolism and antiviral immunity via conserved palmitoylation. However, they differ in substrate specificity, tissue expression, and disease association. Palmitoyltransferase family *ZDHHC11* positively regulates DNA virus-triggered signals: its overexpression activates the IFN-β promoter, while its deficiency impairs HSV-1-induced antiviral gene transcription. Mechanistically, it promotes *IRF3* recruitment to MITA and mediates MITA-dependent DNA virus-targeted innate immunity (Liu et al., 2018). Research on the interaction between *ZDHHC11* and HPV genes remains limited, while a potential link exists in immune regulation and protein modification, direct evidence is lacking. *ZDHHC11* and *ZDHHC11B* should be regarded as hypothesis-generating targets, whose potential functions in modulating cervical microenvironment and interferon therapy responses merit further investigation. Future studies combining molecular biology and virology methods should clarify *ZDHHC11* in HPV infection and carcinogenesis, which may offer new targets for HPV-related disease treatment.

Transcriptome pairwise comparisons showed *TRAF3IP3* were downregulated in clearance patients but upregulated in persistence ones. Previous study showed that *TRAF3IP3* regulates type I interferon responses and impacts antiviral innate immunity (Deng et al., 2020). In addition, our results show consistent expression trends between *TRAF3IP3* and *ZBP1.* Prior studies show ZBP1, induced during coronavirus infection, impairs IFN therapy efficacy by driving inflammatory cell death and lethality. We thus hypothesized ZBP1 also influences differential responses to local interferon therapy (Karki et al., 2022). *TRAF3IP3* and *ZBP1* are efficacy-related differential markers in both independent and self-paired comparisons. With limited research on their interaction with HPV, future work will build a multi-dimensional experimental system (gene regulation, viral replication, immune signals) to explore their role in HPV clearance.

Within the cervical immune microenvironment, cytokines mediate the immune system’s key role in HR-HPV infection. After HR-HPV particles are phagocytosed by dendritic cells (DCs) and transported to local lymphoid tissues, DCs secrete pro-inflammatory cytokines (IL-1α, IL-1β, IL-6, TNF-α, IL-12) to activate cellular and humoral immunity, supporting HR-HPV clearance (Ntuli et al., 2022). Our lab’s prior studies show systemic cytokine profiles do not reflect cervical local microenvironment immunity, due to marked differences in local cytokine expression (Ji et al., 2026). Cytokine values here were cube-root transformed, but this did not affect the consistency of the original statistical comparison results. Notably, IL-4 and IL-5 were extremely low, while IL-6, IL-8, IL-1β *etc.* were highly expressed. This provides a reference for future selection of cytokine targets in cervical secretions. Previous studies indicates that TRAF3IP3 negatively correlates with IFN-β, as its overexpression inhibits IFN-β production while genetic deficiency enhances IFN-β induction, and this regulatory effect is mediated through the TRAF3IP3-TBK1-IRF3 axis by promoting TBK1 degradation to suppress downstream IFN-β output (Deng et al., 2020). ZBP1 has been shown to positively correlate with IL-8, IFN-α/β, and other key cytokines, exerting its function by driving cytokine production via the ZBP1-NF-κB signaling axis (Li et al., 2024). Notably, despite their distinct molecular targets and signaling cascades, both TRAF3IP3 and ZBP1 play pivotal roles in modulating IFN and inflammatory cytokine networks, with IFN-β serving as a central mediator that indirectly shapes the downstream cytokine milieu, highlighting the complexity of innate immune regulation and providing a foundation for further exploring the coordinated effects of these molecules in antiviral and inflammatory responses. Our study also found increased IL-2 and IL-8 in HPV-clearance patients while IL-12p70 increased in HPV-persistence patients, reflecting the cervical microenvironment’s complex regulation.

Our study has several limitations that should be considered, alongside its notable strengths. While the sample size was relatively small (only 29 patients), which may restrict the generalizability of the findings, this limitation was partially mitigated by our paired sample design—we collected samples from each patient at two time points, and both subgroup analyses of a subset of these paired samples and full analyses of all 29 paired samples yielded nearly consistent results, with transcriptome data showing highly similar pathway enrichment patterns, reflecting to some extent the consistency of the observed phenomena and endowing the study with certain research value, We also conducted internal and external validation for the identified key signature genes. However, we did not validate the specific genes associated with passway of differentiation, keratinization, and development; We will conduct further supplementary validation when opportunities arise in the future. In terms of sample collection, we used cotton swabs to gather cervical secretions, a method simpler to implement than vaginal lavage but one that might introduce minor sampling errors, though this risk was reduced by having the same clinician perform all collection procedures, minimizing variability caused by inter-operator differences. Complementing these efforts to address limitations, our study also had distinct strengths that is we employed an immunofluorescence assay to detect 12 cytokines, a technique with high sensitivity and accuracy that ensured the reliability of cytokine-related data. Additionally, the research integrated three interconnected levels—microbial, host transcriptome, and cervical microenvironment—to characterize differential response markers to local interferon-based therapy, resulting in a more systematic and comprehensive investigation of the topic.

## Data Availability

The data presented in this study have been deposited in the Sequence Read Archive (SRA) repository under the BioProject ID: PRJNA1428916 (https://www.ncbi.nlm.nih.gov/sra/PRJNA1428916).
